# Identification of Imprinted Genes Based on Homology: An Example of *Fragaria vesca*

**DOI:** 10.3390/genes12030380

**Published:** 2021-03-08

**Authors:** Yaping Liu, Xiaotong Jing, Hong Zhang, Jinsong Xiong, Yushan Qiao

**Affiliations:** Laboratory of Fruit Crop Biotechnology, College of Horticulture, Nanjing Agricultural University, No. 1 Weigang, Nanjing 210095, China; 2017104020@njau.edu.cn (Y.L.); 2018204005@njau.edu.cn (X.J.); 2016204012@njau.edu.cn (H.Z.); jsxiong@njau.edu.cn (J.X.)

**Keywords:** imprinted genes, conservation, *Fragaria vesca*, expression

## Abstract

Genomic imprinting has drawn increasing attention in plant biology in recent years. At present, hundreds of imprinted genes have been identified in various plants, and some of them have been reported to be evolutionarily conserved in plant species. In this research, 17 candidate genes in *Fragaria vesca* were obtained based on the homologous imprinted genes in *Arabidopsis thaliana* and other species. We further constructed reciprocal crosses of diploid strawberry (*F. vesca*) using the varieties 10-41 and 18-86 as the parents to investigate the conservation of these imprinted genes. Potentially informative single nucleotide polymorphisms (SNPs) were used as molecular markers of two parents obtained from candidate imprinted genes which have been cloned and sequenced. Meanwhile, we analyzed the SNP site variation ratios and parent-of-origin expression patterns of candidate imprinted genes at 10 days after pollination (DAP) endosperm and embryo for the hybrids of reciprocal cross, respectively. A total of five maternally expressed genes (MEGs), i.e., *FvARI8*, *FvKHDP*-2, *FvDRIP2*, *FvBRO1*, and *FvLTP3*, were identified in the endosperm, which did not show imprinting in the embryo. Finally, tissues expression analysis indicated that the five imprinted genes excluding *FvDRIP2* mainly expressed in the endosperm. This is the first report on imprinted genes of *Fragaria*, and we provide a simple and rapid method based on homologous conservation to screen imprinted genes. The present study will provide a basis for further study of function and mechanism of genomic imprinting in *F. vesca*.

## 1. Introduction

Genomic imprinting is a phenomenon in which paternal and maternal alleles are differentially expressed in the offspring depending on their parental origin [1]. Therefore, based on their parent-of-origin manner, imprinted genes are divided into two groups, maternally expressed genes (MEGs) and paternally expressed genes (PEGs). Until now, genomic imprinting has been identified in fungi, mammals, and flowering plants [2]. Gene imprinting in flowering plants occurs mainly in the endosperm, and only a few imprinted genes are described in the embryo [3,4,5,6,7]. The endosperm and embryo of flowering plants are both derived from a double fertilization event: the egg cell (1n) and central cell (2n) fuse with two sperm cells (1n) to form the diploid embryo (2n) and the triploid endosperm (3n), composed of two maternal and one paternal genome copy, respectively [8,9,10].

It has been demonstrated that genomic imprinting is an epigenetic modification process which includes DNA methylation and trimethylation of histone H3 lysine 27 (H3K27me3) [11], and many genes involved in this process are imprinted ones. For example, in *A. thaliana*, *MEDEA (MEA)*, and *FERTILIZATION-INDEPENDENT ENDOSPERM (FIE)*, the components of polycomb repressive complex 2 (PRC2) that mediate H3K27me3 were found maternally expressed during endosperm development [12,13,14,15,16]. Interestingly, studies on maize found that the homologs of *MEA* and *FIE* were also imprinted [17,18]. Moreover, in rice, *OsFIE* was also identified as an imprinted gene [19]. Besides the genes of PRC2, *YUCCA10, VIM5, VIM1*, and *ARID-BRIGHT DNA binding domain* from *A. thaliana* and their homologs in rice and maize, *29765.m000727* from castor bean and its homolog in *A. thaliana*, rice and maize, have also been identified as imprinted genes [20,21]. These studies indicated that genomic imprinting is evolutionarily conserved in flowering plants.

In this study, based on evolutionary conservation of genomic imprinting across plant species, we constructed a reciprocal cross of wild diploid strawberry *F. vesca* to test the conservation of PRC2-related genes *MEA*, *FIE*, and 16 other conserved imprinted genes in strawberry. By analyzing the imprinting status of candidate conserved imprinted genes based on SNP site variation ratios and parent-of-origin expression patterns, we found that five genes were conserved imprinted in strawberry endosperm. Moreover, the tissue-specific expression characteristics of these five genes are similar to the reported imprinted genes in other plants [7,22]. Our findings not only give us a preliminary understanding of strawberry genomic imprinting, but also provide evidence of the conservation of imprinted genes. It will help us to better explore the function and mechanisms of imprinted genes.

## 2. Results

### 2.1. Identification of Candidate Imprinted Genes

According to the conserved imprinted gene information from *A. thaliana*, rice, and maize, 11 candidate genes were obtained, namely, *FvFIE*, *FvYLS9*, *FvMLP*, *FvUNP*, *FvARI8*, *FvKH*, *FvCAL*, *FvUNP1*, *FvMIA40*, *FvAMI*, and *FvTAR4*. BioXM software was used to analyze the similarity of the protein sequences of *A. thaliana* and *F. vesca*, and the amino acid sequence similarities were 75.48%, 69.3%, 71.25%, 50.53%, 74.5%, 85.92%, 50.68%, 57.38%, 46.88%, 60.57%, and 43.5%, respectively. Using the same method, four candidate genes similar to conserved imprinted genes of *A. thaliana*, rice, and sorghum were obtained from *F. vesca*, namely, *FvVIP2*, *FvKHDP-2*, *FvDRIP2*, and *FvBRO1*. Besides, *FvLTP3* and *FvYLS3* were potential orthologs of conserved imprinted genes from *A. thaliana* and castor bean. Homologous protein of *MEA* was not found in the *F. vesca*. Detailed information is shown in Table 1.

### 2.2. SNP Information of Candidate Imprinted Genes in Parents

In total, 17 homologous genes were obtained by BLASTP from *F. vesca*, and the coding sequences (CDs) of candidate genes were obtained by RT-PCR cloning and sequencing. SNPs were analyzed based on the sequencing results of 17 candidate genes from 10-41 and 18-86 leaves. Among the 17 genes we amplified, 10 candidate genes (*FvFIE*, *FvYLS9*, *FvMLP*, *FvUNP*, *FvKH*, *FvVIP2*, *FvYLS3*, *FvUNP1*, *FvMIA40*, and *FvAMI*) lacked SNP sites between two *F. vesca* ecotypes, which means that there is no difference among these sequences between the parents. The remaining seven candidate genes have SNPs between parental ecotypes, of which *FvARI8* has 1 SNP site, *FvBRO1* has 2, *FvKHDP-2* has 19, *FvDRIP2* has 17, *FvLTP3* has 3, *FvTAR4* has 11, and *FvCAL* has 2 (Table S1).

### 2.3. Biallelic Expression of FvTAR4 and FvCAL Genes in Endosperm

*FvTAR4* and *FvCAL* genes have 11 and 2 SNPs, respectively. Amplification and sequencing results showed that there are two types of expression at the SNP site of the hybrid endosperms. At position 314 of *FvTAR4*, the SNP site has two different bases, A and T (Figure S1A). Similarly, *FvCAL* has A and G at position 406 (Figure S1B), and it was estimated that the base variation ratio of its SNP site is close to 2: 1, which is in line with the expected value. Thus, we speculated that *FvTAR4* and *FvCAL* genes are not imprinted genes.

### 2.4. Five Genes Show Imprinting in the Endosperm of Wild Strawberry

**Figure 1:**
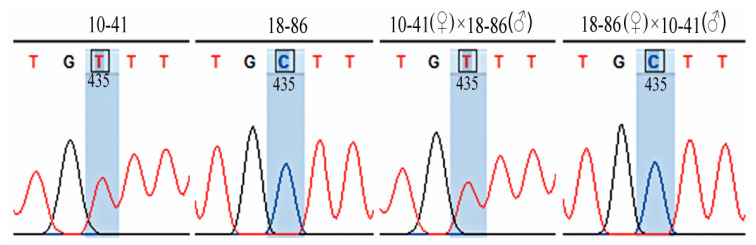
Monollelic expression of *FvARI8* in reciprocal cross endosperm of two *Fragaria vesca* ecotypes. Sequencing showing that *FvARI8* is a monollelic-specific gene, and expressed in a maternal parent-of-origin-dependent manner.

There is only one SNP site in the coding region of *FvARI8* in the 10-41 and 18-86 parents. The expression pattern of this SNP site in reciprocal cross endosperm is shown in Figure 1. In the hybrid endosperm of 10-41 maternal, the SNP site of *FvARI8* at the same position is the same as the parent 10-41; and in the hybrid endosperm of the 10-86 maternal parent, the SNP site at the same position is the same as 18-86. In other words, *FvARI8* showed monoallelic expression in a parent-of-origin-dependent manner, indicating this gene is not only a MEG but also a binary imprinting. Expression patterns of *FvKHDP-2*, *FvDRIP2*, *FvBRO1,* and *FvLTP3* genes in the hybrid endosperm at the SNP site are shown in Figures S2–S5 and their expression patterns are the same as *FvARI8*, which confirmed that gene imprinting was conserved in some plants.

### 2.5. Candidate Imprinted Genes Show Non-Imprinting in the Embryos

We tested the seven candidate genes with SNPs between two ecotypes of *F. vesca*, and verified the imprinted genes in the reciprocal cross embryo of 10 days after pollination (DAP). Results are shown in Figure S6. Alleles of the maternal and paternal of *FvARI8*, *FvKHDP-2*, *FvDRIP2*, *FvBRO1*, *FvLTP3*, *FvCAL*, and *FvTAR4* genes are all expressed in the embryos of the hybrid combinations 18-86 × 10-41 and 10-41 × 18-86, and base variation ratio at the SNP site is close to the expected value of 1:1. We surmised that these genes are non- imprinted genes in reciprocal cross embryo.

### 2.6. Expression Patterns of Imprinted Genes in Different Tissues

**Figure 2:**
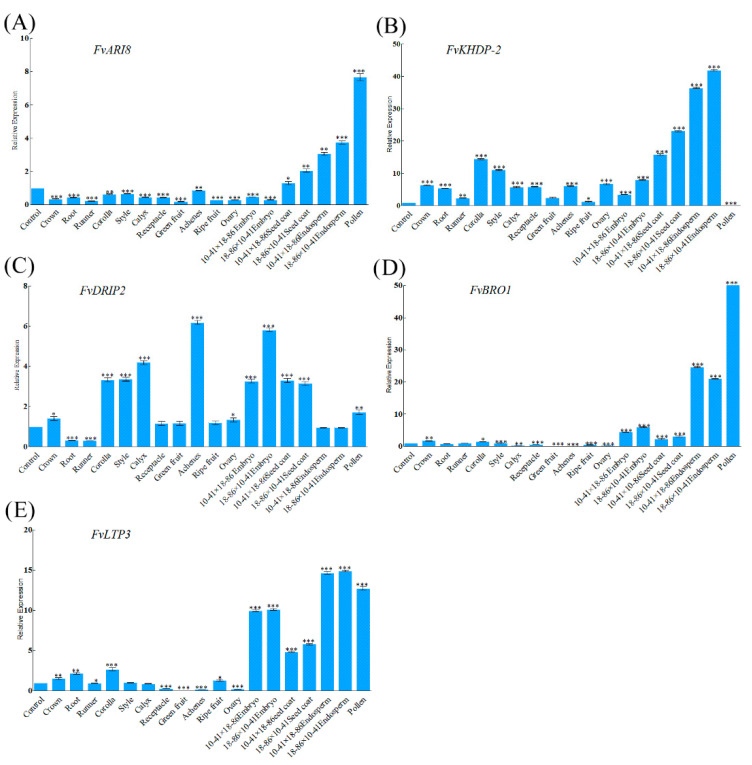
Expression of five imprinted genes in different tissues of *F. vesca*. Error bars represent standard deviation (SD). Columns with asterisks indicate significant difference: * represents *p* ≤ 0.05, ** represents *p* ≤ 0.01, *** represents *p* ≤ 0.001. *FvARI8*(**A**), *FvBRO1*(**D**), *FvKHDP-2*(**B**), and *FvLTP3*(**E**) showed a similar expression pattern, whereas *FvDRIP2*(**C**) has a lower expression level in endosperm and pollen.

In order to investigate whether five MEGs have specific expression characteristics in endosperm, qRT-PCR was used to analyze expression patterns of these genes in different tissues of *F. vesca*. Expression patterns of *FvARI8* and *FvBRO1* are similar, all showing a lower expression level in nutritional tissues, and some reproductive tissues such as style, fruit, and ovary, but higher expression in endosperm and pollen (Figure 2A,D). *FvBRO1* has a higher expression level in the hybrid endosperm and embryo tissues of 10 DAP compared with other tissues except pollen, and a lower expression in style. Expression of *FvBRO1* in green fruit, ripe fruit, receptacle, and achene is extremely low, indicating that it may be less important in the development of fruit (Figure 2D). *FvKHDP-2* shows weakly expressed in pollen (Figure 2B). Tissue-specific higher expression of *FvDRIP2* was observed in achenes and embryo (Figure 2C). The low expression pattern of *FvLTP3* in nutritional tissues is similar to that of *FvARI8* and *FvBRO1*, whereas *FvLTP3* shows a higher expression level in embryo, endosperm, and pollen (Figure 2E). In addition, we found all of the imprinted genes have a low expression in root and runner.

## 3. Discussion

The phenomenon of genomic imprinting has been widely observed in animals and plants. In plants, the imprinted gene was first identified in maize by phenotypic identification methods [23]. However, only a handful of imprinted genes had been identified in plants until the emergence of next-generation sequencing technology. At present, more and more imprinted genes have been identified and characterized in other plant species, including *A. thaliana* [4,5,7,24,25], rice [26,27,28], maize [20,22,29], sorghum [30], castor bean [21], wheat [31], tomato [32,33], *Capsella rubella* [34], and *Brassica* [35,36]. Further study indicated that many imprinted genes were found to be conserved among different species. For example, 24 imprinted genes in *A. lyrata* were found to be imprinted in *A. thaliana* [25], and 55.6% imprinted genes are reported as conserved imprinted genes in hexaploid wheat and its close relative tetraploid [31]. Chen et al. [37] compared the imprinted genes obtained in rice with those in *A. thaliana*, maize, and sorghum, and found that several of these genes were conserved imprinted genes in these species. They further identified eight homologous imprinted genes in barley (*Hordeum vulgare*) through the conservative imprinted genes from rice.

In this study, *MEA*, *FIE*, and 16 conserved imprinted genes in *A. thaliana* and their homologs of four other plants were used as inquiry genes to screening imprinted homologs in diploid strawberry *F. vesca*. As shown in Table 1, all genes except *MEA* have homologs in *F. vesca*, indicating that this is a feasible approach to identify imprinted genes in plants. To verify that the 17 candidates were true imprinted genes, we cloned and sequenced the CDs of 17 genes from the respective parents. Sequence analysis indicated that 10 of the 17 candidates lacked SNPs between parents. Therefore, it is difficult to determine whether these 10 genes are imprinted genes. The remaining seven candidate genes that contained more than one SNPs were further analyzed in both endosperm and embryo. Results indicated that five of the seven genes, i.e., *FvARI8*, *FvKHDP-2*, *FvDRIP2*, *FvBRO1*, and *FvLTP3*, were imprinted in endosperm. Interestingly, these seven genes all showed non-imprinting expression in reciprocal cross embryo. These results were consistent with research results that indicate that gene imprinting in flowering plants mainly occurs in endosperm [3,4,5,6,7].

The identification of imprinted genes in the endosperm and embryo can be confounded by maternal tissues [38,39] and parent-of-origin effects [40]. The seed coat, a maternal tissue that surrounds and protects the seed, can lead to false-positive MEGs. To reduce false-positive MEGs, Pignatta et al. 2014 [7] censored the genes with expression that was more than twice as high in seed coat relative to endosperm during RNA-seq analyses. In this study, we also analyzed the expression of imprinted genes in the seed coat (Figure 2), the results showed that they have a lower expression level in seed coat relative to endosperm excluding *FvDRIP2*. In previous studies, the imprinted genes regulated by specific epigenetic marks, such as *MEA* [14,15,16], *FIS2* [15], *LORELEL* [41], *NUWA* [42], and *Mez1* [17], etc., have a monoallelic expression. Similarly, five imprinted genes identified in this study also show a monoallelic expression in a parent-of-origin-dependent manner. However, examples of monoallelic expression of non-imprinted genes have also been reported, which was contribute to phenotypic diversity in poplar [43], barley [44], rice [45], and *A. thaliana* [46]. In addition, maternal effects can lead to the appearance of a parent-of-origin effect because of the deposition of mRNA from gametophytic in the fertilized egg cell (zygote) or fertilized central cell (endosperm) during early seed development, which will increase contamination of RNA-seq analyses [38]. In this regard, sequencing cannot distinguish imprinting from contamination or parent-of-origin effects, hence we need further research to prove that these genes show a monoallelic expression in a parent-of-origin-dependent manner are modified by specific epigenetic marks.

Further analysis indicated that the five strawberry endosperm imprinted genes were MEGs; however, their homologs in other plants belong to PEGs. For example, *FvKHDP2* and its homolog in *A. thaliana* are MEG, while its ortholog in rice and *Sorghum bicolor* is PEG, indicating that although they are conservative imprinted genes, the regulatory mechanism of imprinted expression in different species may be different. MEGs and PEGs differ in their targeting by 24-nt small RNAs and asymmetric DNA methylation, suggesting different mechanisms establishing DNA methylation at MEGs and PEGs [34,47,48]. Batista and Köhler [11] thought that MEGs were dependent on parental DNA methylation asymmetries, and that PEGs were dependent on parental asymmetric DNA methylation and H3K27me3.

Among the five imprinted genes, *FvARI8* and *FvDRIP2* belong to the E3 ubiquitin ligase RING protein family; *FvARI8* is structurally similar to *AtARI8* in *A. thaliana*, with a RING1-IBR-RING domain, a supercoiled domain and a leucine-rich region at the C-terminus, which plays an important role in maintaining protein binding; *AtARI8* is expressed in stems, leaves, flowers, and silique [49]; and *FvARI8* is also expressed in these tissues, which may have similar functions, but we have not found any functional studies. Amino acid sequences of *FvDRIP2* and *Vigna unguiculata VuDRIP* are 48.85% similar. *VuDRIP* interacted with *VuDREB2A* was detected by yeast two hybrids, *DRIP* negatively regulates *DREB2A*, which reduces the expression of *DREB2A* under non-stress conditions, thereby reducing the metabolic burden [50]. *FvDRIP2* may also participate in stress-related regulation. *FvKHDP-2* contains the KH domain. Proteins containing KH domains perform multiple cellular functions. KH domain is essential for establishing a post-transcriptional regulatory network, and it also has E3 ubiquitin ligase activity [51,52,53]. *FvBRO1* is a member of the heavy-metal-associated protein family. Studies have shown that members of this family are mainly involved in the process of heavy metal accumulation in *A. thaliana* [54]. *FvLTP3* is a non-specific lipid transfer protein, it plays an important role in plant defense and should also be applied to abiotic stresses such as drought, cold, and salt [55,56].

The five strawberry MEGs’ expression characteristics are similar to the imprinted gene expression patterns in maize [22] and *A. thaliana* [7], that is, they are both highly expressed in the endosperm and may be involved in the seed development. Tissue expression specificity analysis also found that identified imprinted genes have expression levels in various tissues, indicating that imprinted genes play a role in other tissues in addition to their role in endosperm development. Similarly, 67% of MEGs in castor bean were found to be expressed in other tissues [21], which suggests that imprinted genes also play a role in the development of other tissues. Future study will be undertaken to characterize the functions of these strawberry imprinted genes. In summary, this simple and rapid method of identifying imprinted genes based on homology is feasible. Although the number of imprinted genes obtained is limited, as more imprints are discovered, this method will show greater power. To our knowledge, this is the first to find imprinted genes in the endosperm in the Fragaria.

## 4. Materials and Methods

### 4.1. Plant Materials

Wild *F. vesca* ecotype 10-41 (collected from Europe) and 18-86 (collected from Tianshan, Fukang, Xinjiang, China) were planted in the greenhouse at Baima Teaching and Scientific Research Base of Nanjing Agricultural University, Nanjing, China. Unopened flowers were emasculated; then, the pollinated flowers were bagged at the beginning of flowering in April.

Developing achenes from reciprocal cross 18-86 and 10-41 at 10 DAP were collected. Embryos, endosperms, and seed coats were manually dissected and washed using tissue separation buffer (5 mM MES (Methyl ethanesulfonate), 0.3 M Sorbitol, pH5.7). Then, we collected the 10-41 naturally growing root, runner, crown, leaf, green fruit (18 DAP), ripe fruit (30 DAP), ripe achene, style (including stigma), ovary (excluding stigma), corolla, receptacle, calyx, and pollen. All tissues were immediately frozen by liquid nitrogen and then stored at −80 °C until use.

### 4.2. Acquisition of Candidate Imprinted Genes

Some conserved imprinted genes have been reported in plants, was listed in Table 2. Using the protein sequences encoded by imprinted genes in *A. thaliana* as the query sequences, BLASTP was performed in the *F. vesca* protein database based on National Center for Biotechnology Information (NCBI) (E-value < 10 × 10^−10^). Multiple sequence alignment of proteins was performed between species by using BioXM (https://cbi.njau.edu.cn/BioXM/ (accessed on January 2014)) and MEGA-X [57] to further confirm their similarity.

### 4.3. RNA Isolation and Cloning of Candidate Imprinted Genes

Total RNA from different tissues was extracted using an RNA extraction kit (Tiangen, Beijing, China), and RNAs were reverse-transcribed into complementary DNA (cDNA) using the PrimeScript RT reagent kit (TaKaRa, Dalian, China). According to the candidate gene sequences from NCBI, Primer 5.0 software was used to design specific primers among the CDs (Table 3). The cDNAs of leaves from the parents, endosperm, and embryo of the reciprocal cross between *F. vesca* ecotypes 10-41 and 18-86 at 10 DAP were performed using PCR amplification with primers in the CDs. The PCR program was as follows: 1 cycle of 5 min at 98 °C; 35 cycles of 30 s at 94 °C, 30 s at 55 °C, 1 min at 68 °C; and a final extension for 10 min at 68 °C. All PCR products were analyzed by agarose gel electrophoresis, and the target band was recovered using an AxyPrep DNA gel recovery kit (Axygen, Union City, CA, USA). Then, the target fragment was ligated into a pCloneEASY Blunt Vector Kit (TransGen Biotech, China) and transformed into *Escherichia coli* DH5α (Tsingke Biotechnology, China). Finally, 20 single colonies for each fragment were picked and grown overnight at 37 °C for Sanger sequencing. Sanger sequencing was performed by Tsingke Biotechnology Ltd. (Beijing, China).

### 4.4. Identification of Imprinted Genes in Wild Strawberry

Potentially informative SNP sites were obtained by aligning the Sanger sequencing data from leaves of 10-41 and 18-86 with the BioXM (https://cbi.njau.edu.cn/BioXM/ (accessed on January 2014)) and MEGA-X [57]. Candidate imprinted genes with pure SNP site were retained, otherwise it would be eliminated. Then, we analyzed SNP information from endosperm and embryo of reciprocal cross to observe the expression manners of alleles based on their parent of origin. Finally, expression ratios of maternal and paternal alleles were calculated for the genes that had SNP sites in endosperm and embryo tissue according to the method of Liu and Qian [59] and ImageJ software [60]. The expression ratio of maternal and paternal alleles is theoretically 2:1 and 1:1 in hybrid endosperm and embryo, respectively [61,62]. In the reciprocal crosses endosperm, MEG was a defined gene with a ratio higher than 4m:1p, and PEG was a defined gene with a ratio higher than 2p:1m. We defined MEG and PEG as being higher than the ratio 3:1 (3m:1p, 3p:1m, respectively) in the reciprocal crosses embryo.

### 4.5. Expression Profiles Analysis of Imprinted Genes

RT-qPCR was performed using an ABI 7300 Real-Time PCR System (Applied Biosystems, Foster City, CA, USA) and SYBR Green Real-time PCR Master Mix (Toyobo, Osaka, Japan). A total reaction volume (20 μL) comprised 10 μL SYBR Green Master Mix, 8.4 μL ddH_2_O, 1 μL cDNA sample (equivalent to 100 pg of total RNA), and 0.3 μL of each primer (the final concentration of all primers was 10 μM). The primers involved are listed in Table 4. The reactions were incubated at 95 °C for 4 min, followed by 40 cycles at 94 °C for 20 s, 62 °C for 20 s, and 72 °C for 40 s (extending and gathering the fluorescent signal). Four technical replicates were performed for three biological replicates of each sample. Quantitative analysis of gene expression was performed using the 2^−ΔΔCT^ [63] method, and SPSS software version 25.0 was used for statistical analysis.

## Figures and Tables

**Table 1:** Details of 17 candidate imprinted genes of *Fragaria vesca*.

Query Sequence	BLASTP Result	Gene name	E-Value	Similarity (%)	Length of Coded Proteins/aa
*AtMEA*	Not found				
*AtFIE*	XP_004294343.1	*FvFIE*	0	75.48	370
AT5G22200	XP_004303497.1	*FvYLS9*	0	69.3	210
AT1G24020	XP_004297655.1	*FvMLP*	0	71.25	157
AT2G27385	XP_004291583.1	*FvUNP*	1 × 10^−42^	50.53	178
AT2G31510	XP_004302175.1	*FvARI8*	0	74.5	596
AT3G08620	XP_004302513.1	*FvKH*	0	85.92	282
AT5G10140	XP_004306305.1	*FvCAL*	1 × 10^−20^	50.68	219
AT1G07705	XP_004294625.1	*FvVIP2*	4 × 10^−137^	75.75	664
AT2G03110	XP_011467948.1	*FvKHDP-2*	0	50.98	549
AT3G23060	XP_004289907.1	*FvDRIP2*	0	41.41	426
AT4G16380	XP_004293392.1	*FvBRO1*	1 × 10^−30^	49.63	267
AT1G62790	XP_004298676.1	*FvLTP3*	1 × 10^−17^	45.45	151
AT1G62790	XP_011464483.1	*FvYLS3*	2 × 10^−32^	44.19	169
AT3G49540	XP_004307185.1	*FvUNP1*	1 × 10^−22^	57.38	185
AT3G49540	XP_004300088.1	*FvMIA40*	5 × 10^−16^	46.88	144
AT3G49540	XP_004290526.1	*FvAMI*	0	60.57	603
AT1G23320	XP_004290481.1	*FvTAR4*	2 × 10^−74^	43.5	485

**Table 2:** Conserved imprinted genes between *A. thaliana* and four other species.

Imprinted Genes of *A. thaliana*	Conserved Imprinted Genes of Four Species ^a^				
	*Oryza sativa*	*Zea mays*	*Sorghum bicolor*	*Ricinus communis*	References
*AtMEA ^m^*		*ZmMez1 ^m^*			[14,17]
*AtFIE ^m^*	*OsFIE1^m^*	*ZmFIE1 ^m^*			[15,18,19]
*AT5G22200 ^m^*	*LOC_Os04g58860 ^m^*	*GRMZM2G358540 ^m^*			[37]
*AT5G22200 ^m^*	*LOC_Os11g05860 ^m^*				[37]
*AT1G24020 ^m^*	*LOC_Os04g39150 ^m^*	*GRMZM2G102356 ^m^*			[37]
*AT2G27385 ^m^*	*LOC_Os10g05750 ^m^*	*GRMZM2G003909 ^m^*			[37]
*AT2G31510 ^m^*	*LOC_Os04g41470 ^p^*	*GRMZM2G006428 ^p^*			[37]
*AT3G08620 ^m^*	*LOC_Os07g12490 ^p^*	*GRMZM2G472052 ^p^*		*29765.m000727 ^m^*	[21,37]
*AT5G10140 ^p^*	*LOC_Os12g10540 ^m^*	*GRMZM2G010669 ^m^*			[37]
*AT1G07705 ^p^*	*LOC_Os02g54120 ^p^*		*Sb04g035110 ^p^*		[37]
*AT2G03110 ^m^*	*LOC_Os10g35220 ^p^*		*Sb07g005630 ^p^*		[37]
*AT3G23060 ^m^*	*LOC_Os12g40790 ^m^*		*Sb08g020500 ^m^*		[37]
*AT4G16380 ^m^*	*LOC_Os05g13940 ^m^*		*Sb08g022000 ^p^*		[37]
*AT1G62790 ^m^*				*30190.m011003 ^m^*	[58]
*AT3G49540 ^m^*	*LOC_Os09g31080 ^m^*	*GRMZM2G129781 ^m^*	*Sb02g003315 ^m^*		[37]
*AT1G23320 ^p^*	*LOC_Os05g07720 ^p^*	*GRMZM2G127160 ^p^*	*Sb09g005080 ^m^*		[37]

^a^, the genes in the same row are conserved with imprinted genes of *A. thaliana*. *^m^*, conserved imprinted gene was identified as maternally expressed gene (MEG). *^p^*, conserved imprinted gene was identified as paternally expressed gene (PEG).

**Table 3:** Primer sequences for cloning candidate genes.

Gene Name	Froward Primer (5′–3′)	Reverse Primer (5′–3′)	Expected Size/bp
*FvFIE*	ATGGCCAAGTTCGCTTTG	TCAAGAATTTTCCATGACATCCC	1113
*FvYLS9*	ATGTCTTCGAAAGACTGCG	TCATATGCTTACCTTGCATCGC	633
*FvMLP*	ATGGCGCCTTCAGATGTTGG	TCAATGAGCGAGGACATAGTC	474
*FvUNP*	ATGGCTACTCTTTCCGGC	TTAGGGTATGCCTATGATAGGGAAG	537
*FvARI8*	ATGGAATCAGAGGACGATTTCG	CTACCGGCGTTGTTGGCA	1791
*FvKH*	ATGTCAGGGTTGTATAATCCC	TCATCGACCTGTTTTGGCAC	849
*FvCAL*	ATGGGAAGAGGGAAGGTGC	CTAAAACAAATTAAGCACTGGATG	660
*FvVIP2*	ATGTCTGGATTACTTAAT	TCAGTGCTGAGGTAACGT	1995
*FvKHDP-2*	ATGGCCGGCCAGAGAAACA	AATGTAACCATAGTTTCTCCGCCG	1647
*FvDRIP2*	ATGGCGAATCAGGTGGTG	GAGGTACAATAATGGCAATGG	1281
*FvBRO1*	ATGGGCGAAAAAAAGGTGACG	TTACATGATGGCGCACGCTT	804
*FvLTP3*	ATGGGTTGCGGCAACATTTC	CTAGTAATACATGACGGAAGCC	456
*FvYLS3*	ATGGCTTCAAAGTGTCTGTT	CTAAAATGCTGCTGCTGGAAT	510
*FvUNP1*	ATGGGTGCTTCCAACTCCAT	CTATTGCTTCCCCTCAGACGAA	558
*FvMIA40*	ATGGGAGGTGCTTCCATCAC	CTAACCTTCTTGCTTGTCAG	435
*FvAMI*	ATGGCGGATCAGGAGGATGA	TCAGACAATACTAGGCGCATCC	1812
*FvTAR4*	ATGGCTAAGCTACAACAAAGCTCC	CTACTTACGTGACTTGCGTCT	1458

**Table 4:** qRT-PCR primer sequence of imprinted gene of *F. vesca*.

Gene name	Froward Primer (5′–3′)	Reverse Primer (5′–3′)	Expected Size/bp
*FvARI8*	CATCCAGTGCCAACCTGAGT	GGCATGTTCGTGGTCAGGTA	152
*FvKHDP-2*	AGCTCAGGGTGGACACAAAG	TCCTCAAAGCCGTTCGTCTC	178
*FvDRIP2*	ATGGCGAATCAGGTGGTGAA	ACACTGCCCAGATCGGTTTT	197
*FvBRO1*	CTTACTGCGCCTCCGTTTCC	AGGGTTAGCAGGAACAACCG	168
*FvLTP3*	ACCTCAACAACACCGACCAA	TGGCGATTCTAACAGCGGAG	153
*EF1-α*	CATGCGCCAGACTGTTGCTGT	GACCGACTCAGAATACTAGTAGC	186

## Data Availability

Data sharing not applicable.

## Supplementary Materials

The following are available online at https://www.mdpi.com/2073-4425/12/3/380/s1, Figure S1: *FvTAR4* and *FvCAL* are biallellic expressions in the endosperms of the hybrid of two *Fragaria vesca* ecotypes. Sequencing of *FvTAR4* and *FvCAL* was used to confirm imprinting status in 10-41 × 18-86 and 18-86 × 10-41. At least ten colonies were sequenced for each amplicon. SNP sites are shaded in blue. *FvTAR4* and *FvCAL* show a biallellic expression, and their variation ratio of SNPs is close to the 2:1 expected ratio in reciprocal cross. Figure S2: Monollelic expression of *FvKHDP-2* in reciprocal cross endosperm of two Fragaria vesca strains. Figure S3: Monollelic expression of *FvDRIP2* in reciprocal cross endosperm of two Fragaria vesca strains. Figure S4: Monollelic expression of *FvBRO1* in reciprocal cross endosperm of two Fragaria vesca strains. Figure S5: Monollelic expression of *FvLTP3* in reciprocal cross endosperm of two Fragaria vesca strains. Figure S6: Biallelic expression of seven candidate genes in reciprocal cross embryo of two *Fragaria vesca* ecotypes. Seven candidate genes with SNPs were cloned in reciprocal cross embryos, and sequencing showed that their variation ratio of SNPs is close to the 1:1 expected ratio. We only show part of the biallelic expression of SNPs, because there is enough to confirm that these candidate genes are not imprinted genes. Table S1: SNP information of candidate gene between *F. vesca* 10-41 and 18-86.
