# Supplementary material for: Identification of Imprinted Genes Based on Homology: An Example of Fragaria vesca
Source: Genes (Basel). 2021 Mar 8;12(3):380. doi: 10.3390/genes12030380 (PMC7999015; doi:10.3390/genes12030380)
Supplement: Supplementary file 1 [file genes-12-00380-s001.pdf]

Table S1. SNP information of candidate gene between *F. vesca* 10-41 and 18-86.

Accession number	Gene name	SNP loci	SNP differential base	
			10-41	18-86
XP_004302175.1	*FvARI8*	435	T	C
XP_004306305.1	*FvCAL*	406	G	A
		426	T	G
		57	C	R
		174	G	T
		192	C	A
		345	G	T
		390	C	T
		447	T	G
		558	C	T
		654	T	C
		657	A	G
XP_011467948.1	*FvKHDP-2*	849	G	A
		879	G	C
		885	C	T
		894	A	T
		1023	C	T
		1041	G	A
		1080	T	C
		1299	C	A
		1437	C	T
		1477	G	A
		69	T	C
		278	G	A
		336	G	A
		374	C	A
		402	A	G
		409	G	A
		510	T	C
		513	A	G
XP_004289907.1	*FvDRIP2*	525	A	T
		549	G	T
		577	A	C
		780	G	A
		792	C	T
		807	C	T
		815	T	C
		902	A	G
		1108	A	G
XP_004293392.1	*FvBRO1*	142	G	A
		678	T	C
		268	G	T
XP_004298676.1	*FvLTP3*	308	C	G
		329	G	T
		314	A	T
XP_004290481.1	*FvTAR4*	354	T	C
		454	G	A

	654	A	G
	807	T	C
	933	A	T
	948	A	G
	1014	G	C
	1083	G	A
	1105	C	T
	1324	A	C

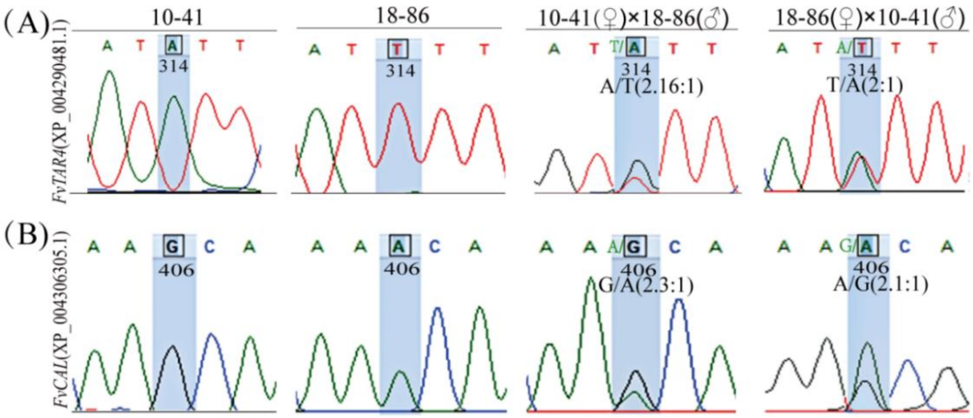

Figure S1. *FvTAR4* and *FvCAL* are biallellic expressions in the endosperms of the hybrid of two *Fragaria vesca* ecotypes. Sequencing of (**A**) *FvTAR4* and (**B**) *FvCAL* was used to confirm imprinting status in 10-41 × 18-86 and 18-86 × 10-41. At least ten colonies were sequenced for each amplicon. SNP sites are shaded in blue. *FvTAR4* and *FvCAL* showing a biallellic expression, and their variation ratio of SNPs is close to the 2:1 expected ratio in reciprocal cross.

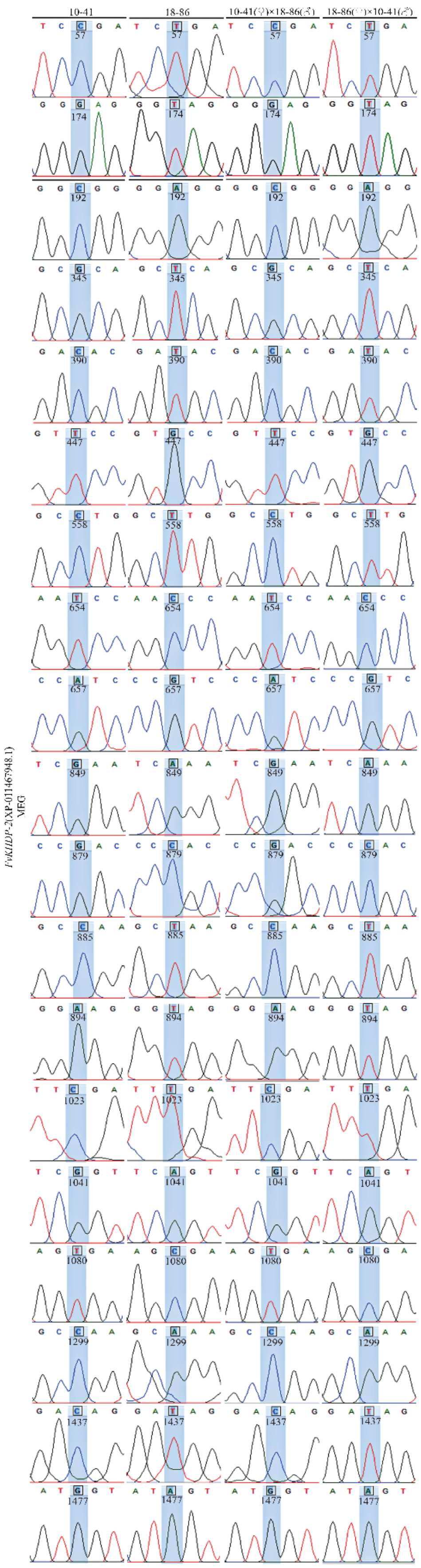

Figure S2. Monollelic expression of *FvKHDP-2* in reciprocal cross endosperm of two Fragaria vesca strains.

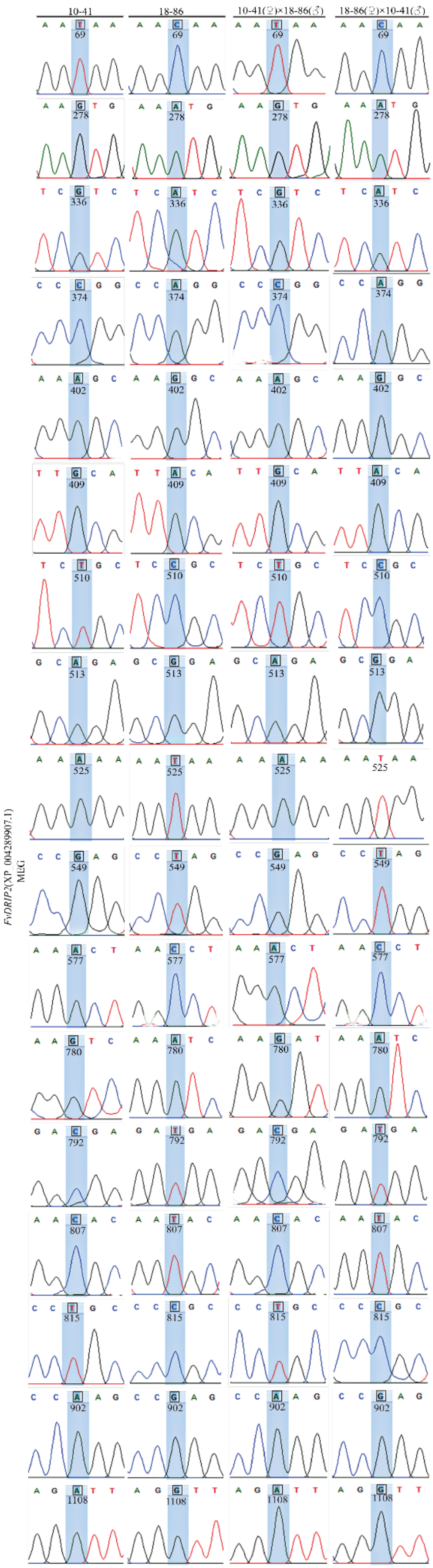

Figure S3. Monollelic expression of *FvDRIP2* in reciprocal cross endosperm of two Fragaria vesca strains.

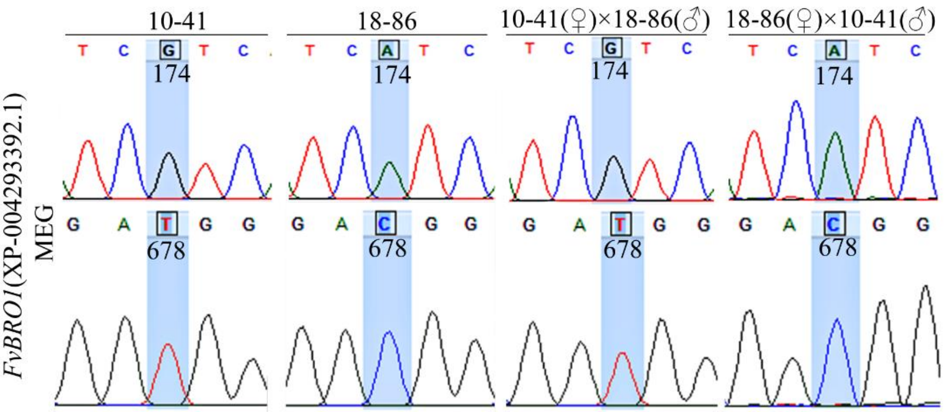

Figure S4. Monollelic expression of *FvBRO1* in reciprocal cross endosperm of two Fragaria vesca strains.

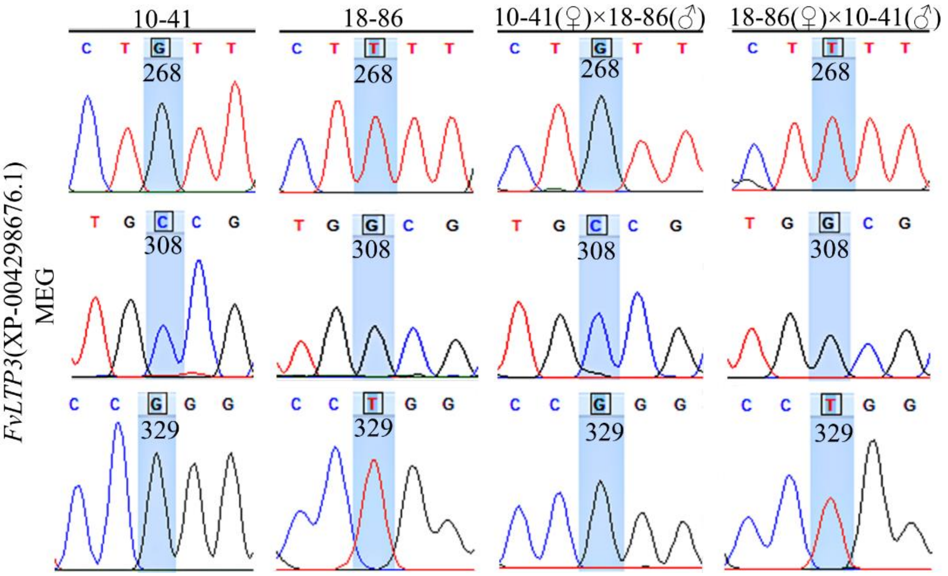

Figure S5. Monollelic expression of *FvLTP3* in reciprocal cross endosperm of two Fragaria vesca strains.

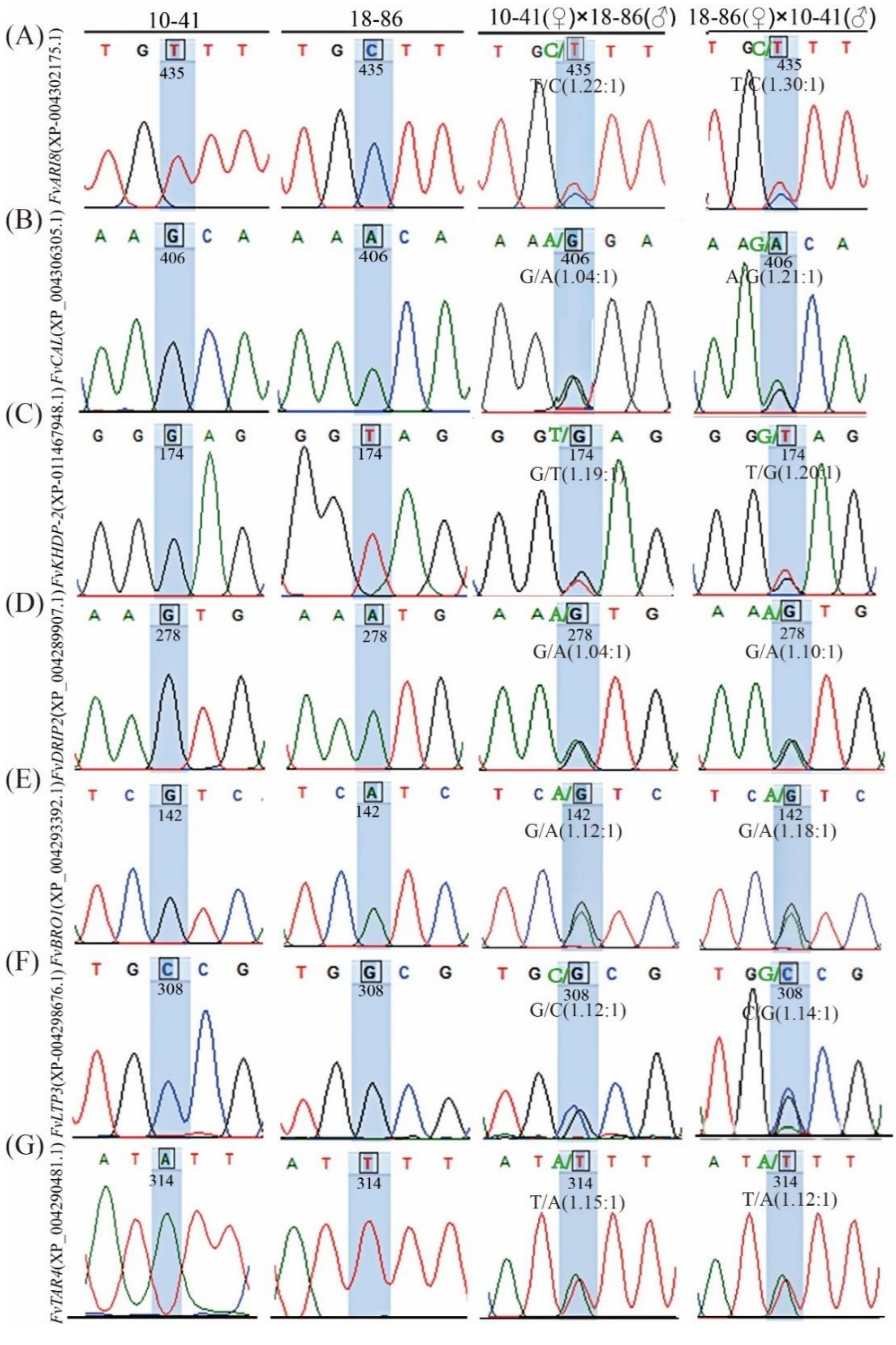

Figure S6. Biallelic expression of seven candidate genes in reciprocal cross embryo of two *Fragaria vesca* ecotypes. (**A**–**G**) Seven candidate genes with SNPs were cloned in reciprocal cross embryos, and sequencing showed that their variation ratio of SNPs is close to the 1:1 expected ratio. We only show part of the biallelic expression of SNPs, because there is enough to confirm that these candidate genes are not imprinted genes.
